# Factors Influencing Pregnancy and Postpartum Weight Management in Women of African and Caribbean Ancestry Living in High Income Countries: Systematic Review and Evidence Synthesis Using a Behavioral Change Theoretical Model

**DOI:** 10.3389/fpubh.2021.637800

**Published:** 2021-02-17

**Authors:** Amanda P. Moore, Angela C. Flynn, Amanda Rodrigues Amorim Adegboye, Louise M. Goff, Carol A. Rivas

**Affiliations:** ^1^Department of Nutrition, Kings College London, London, United Kingdom; ^2^Department of Women and Children's Health, King's College London, London, United Kingdom; ^3^Faculty of Health and Life Sciences, School of Nursing, Midwifery and Health, Coventry University, Coventry, United Kingdom; ^4^UCL Social Research Institute, University College London (UCL), London, United Kingdom

**Keywords:** pregnancy, postpartum, weight, lifestyle, black African, Caribbean, ethnicity

## Abstract

**Background:** Women of black African heritage living in high income countries (HIC) are at risk of obesity and weight-related complications in pregnancy. This review aimed to synthesize evidence concerning attitudes to weight management-related health behaviors in pregnancy and postpartum, in women of black African ancestry, living in high-income countries.

**Methods:** A systematic review of the literature and thematic evidence synthesis using the Capability-Opportunity-Motivation Behavioral change theoretical model (COM-B). Databases searched included MEDLINE, EMBASE, Web of Science, and Scopus. The CASP tool was used to assess quality.

**Results:** Twenty-four papers met the selection criteria, most of which were from the US. Motivational factors were most commonly described as influencers on behavior. Normative beliefs about “*eating for two*,” weight gain being good for the baby, the baby itself driving food choice, as well as safety concerns about exercising in pregnancy, were evident and were perpetuated by significant others. These and other social norms, including a cultural acceptance of larger body shapes, and daily fast food, created a challenge for healthy behavior change. Women also had low confidence in their ability to lose weight in the postpartum period. Behavior change techniques, such as provision of social support, use of credible sources, and demonstration may be useful to support change.

**Conclusions:** The women face a range of barriers to engagement in weight-related health behaviors at this life-stage. Using a theoretical behavior change framework can help identify contextual factors that may limit or support behavior change.

## Introduction

Obesity is a significant global health challenge (1). In most high-income countries (HIC), over 50% of women enter pregnancy with a body mass index (BMI) above the recommended range (2, 3). Excessive gestational weight gain in pregnancy is associated with postpartum weight retention (4, 5), contributing to long-term obesity risk for women (6), particularly when they have a high pre-pregnancy BMI (7, 8). It also increases their risk of gestational diabetes, complications, and mortality in pregnancy (9–12) and impacts on the future health and obesity risks of the offspring (13, 14).

In HIC, women of black African heritage are more likely to enter pregnancy overweight or obese compared with white women (8, 11). Furthermore, black women are four times more likely to remain overweight following pregnancy than white women, with excessive gestational weight gain and postpartum weight retention being key risk factors (15–17).

There is growing evidence to suggest that engagement in optimal diet and physical activity behaviors during pregnancy and the postpartum period can reduce gestational (18–21) and postpartum weight retention (22, 23). However, there is a paucity of studies focusing on modifying lifestyle behaviors in women from ethnic minorities (10, 11, 18, 24).

Improving health behaviors in minority ethnic groups requires healthcare and interventions to be culturally sensitive, taking into consideration beliefs and structural influences on access (25–27). Additionally, it is increasingly recognized that theoretically informed interventions are more likely to show increased efficacy (28), particularly for complex interventions such as those designed to change lifestyle and health behaviors (29, 30). The use of a theoretical framework leads to greater understanding of the influences on health behaviors and provides an opportunity to map these factors to appropriate behavior change techniques, in order to improve the efficacy of interventions (25, 31).

Qualitative methods are a useful approach when exploring attitudes and influences on behavior. Individual studies consider contextually shaped situations, however, they are typically small in size and are setting specific. Synthesizing qualitative studies can lead to a greater understanding of a phenomenon across settings and be of value to researchers and clinicians who are interested in the unique perspectives but who are unlikely to have time to collate all the literature themselves.

**Figure 1:**
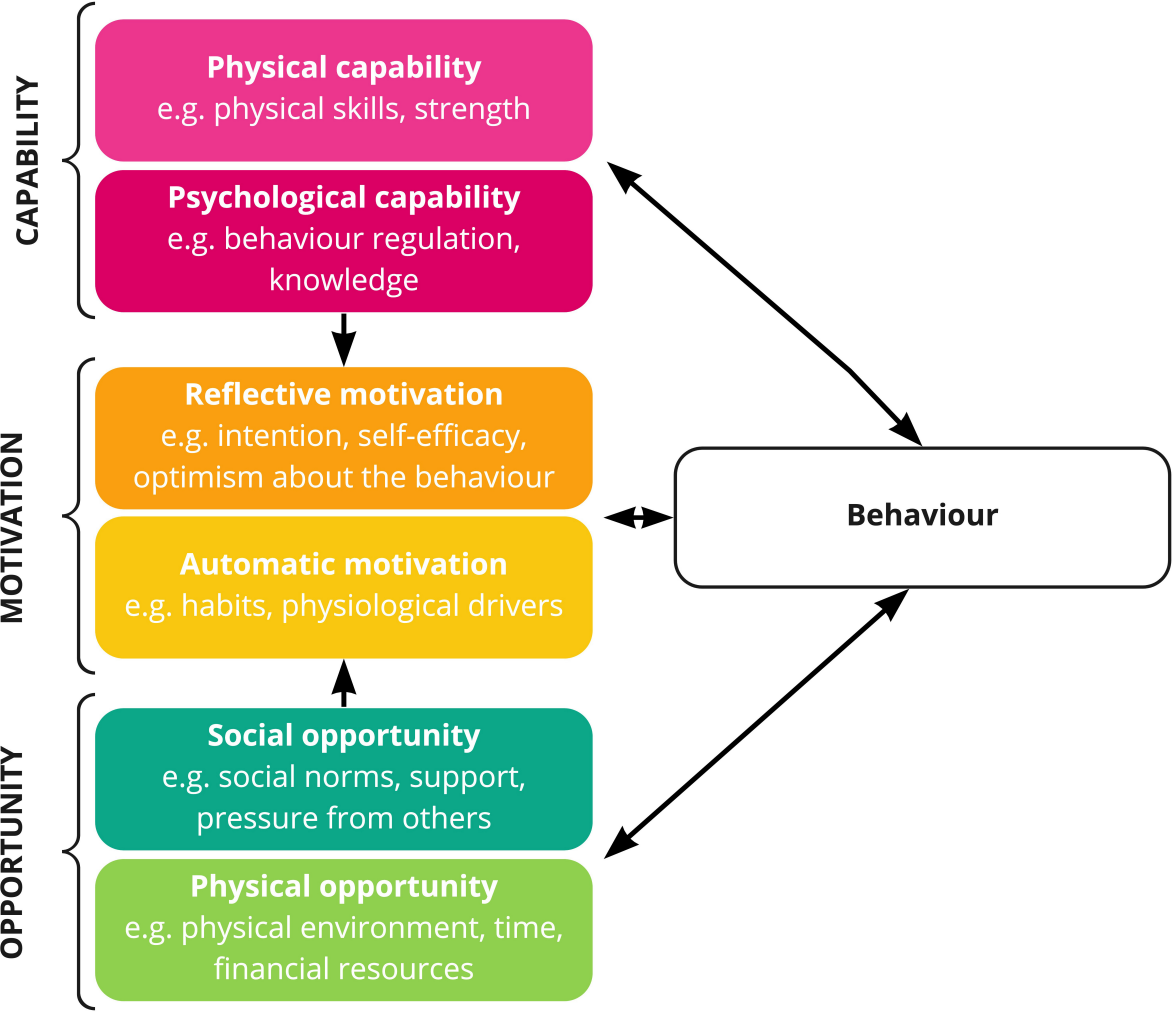
The COM-B framework, modified from Michie et al.

To improve the practical utility of the results, we have applied a theoretical behavioral analysis framework, the Capability-Opportunity-Motivation model of behavior change (COM-B) to our synthesis of the data. In this model the factors that influence behavior are classified according to their influence on Capability (psychological or physical), Opportunity (social or physical), or Motivation (reflective or automatic) (31) (Figure 1). The associated Behavior Change Wheel supports the mapping of the COM-B findings to evidence-based behavior change techniques, linking theory to mechanisms supporting change (32). Underpinning interventions theoretically in this way can improve efficacy and evaluation (33).

This review aims to synthesize the qualitative evidence concerning the attitudes toward and factors influencing engagement in weight management, physical activity and healthy dietary behaviors in the pregnancy and postpartum period in women of African ancestry living in HIC, where there is strong evidence of a disproportionate burden of obesity and weight-related complications associated with pregnancy. The evidence summary will be useful for researchers, healthcare practitioners as well as intervention and health promotion designers.

## Methods

### Reporting

This review paper has been reported in accordance with the Enhancing Transparency of Reporting the Synthesis of Qualitative Research Framework (ENTREQ) and the PRISMA guidelines and is registered on the PROSPERO database for systematic reviews (PROSPERO 2019: CRD42019143056).

### Literature Search

A literature search was conducted on the following electronic databases: MEDLINE, EMBASE, Web of Science, CINAHL, PsycINFO, LILACS, Cochrane Database of Systematic Reviews (CDSR), SCOPUS, and DARE. The search strategy used MeSH index terms and keywords (Table 1, Supplementary File 1). The search strategy was devised for MEDLINE by two members of the review team (AA and AM) and was subsequently modified for the other databases. Each database was searched separately and the results combined. The searches were conducted between 29th June and 18th July 2019 and updated by search alert until 23rd November 2020.

**Table 1:** Summary of sample, phenomenon of interest, design, evaluation, research type (SPIDER) search criteria (34).

**Search term**	**Selection criteria**
Sample	Women of African or Caribbean ancestry, living in high-income countries^*^
Phenomenon of Interest	Pregnancy or postpartum, diet, physical activity, or weight management
Design	Interview, focus groups, case studies, observations, surveys
Evaluation	Attitudes, health knowledge, beliefs, perceptions
Research type	Qualitative, mixed methods

* *European countries, North America, Australia, New Zealand, and Canada*.

### Study Selection

For quality control, two reviewers (AM and GS^1^) independently screened 70% of titles and abstracts for full-text review against the inclusion/exclusion criteria and the final 30% were screened by a single reviewer (AM). Two reviewers independently selected articles for inclusion from the full texts (AA and AM); any differences between reviewers were resolved by a third reviewer (CR).

**The inclusion criteria were**: A qualitative study design (focus groups, ethnography, or interviews) exploring attitudes, behavior or social influences related to exercise, diet, weight management, or body image during pregnancy and postpartum; Incorporating women of black African or Caribbean ancestry (immigrants and long-term settlers (1st−4th generation); HIC setting (Europe, North America, Australia, and New Zealand); Results reporting data for black participants explicitly, or the majority of the sample was of black ethnicity (85%+). **The exclusion criteria included:** Survey methodology; Studies where data concerning exercise, diet, or weight-management was not explicit; Studies focusing on dietary supplement use; Studies where the population criteria or black participant reporting criteria were not met.

Eighty-five percent black participants was chosen as the cut-off by the team for quality reasons when data for black participants was not explicitly reported, such that the themes were likely to be representative of those of black ethnicity. Gestational diabetes (GDM) studies were included if detail regarding diet, physical activity or weight management were reported.

### Data Extraction

Data extraction was carried out according to the published PROSPERO protocol. Information extracted included authors, year of publication, study setting, participant characteristics, data collection method, theoretical framework and author's themes and results. Study results included first- and second-order constructs that were labeled in the results section of the studies, according to reported qualitative synthesis methodology (35).

### Quality Appraisal and Sensitivity Analysis

Studies were appraised for quality using the Critical Appraisal Skills Programme (CASP) tool for qualitative studies (36, 37). No studies were excluded based on the quality appraisal, but a sensitivity analysis was carried out to evaluate the contribution of the weaker studies to the overall findings.

Sensitivity analyses were conducted to assess the impact of study quality and contextual relevance to the review question on the findings (Supplementary File 2).

### Data Synthesis

We employed the thematic synthesis approach as reported by Thomas, Harden et al. (35) using both deductive and inductive methods to synthesize the data. The findings from each study were imported into NVivo as well as other extracted data, e.g., population, setting, topic focus, and pregnancy stage, to allow contextual comparison as the analysis developed. The data were initially coded according to the COM-B framework (31) and then synthesized thematically (35). The coding framework is provided in Supplementary File 3.

## Results

**Figure 2:**
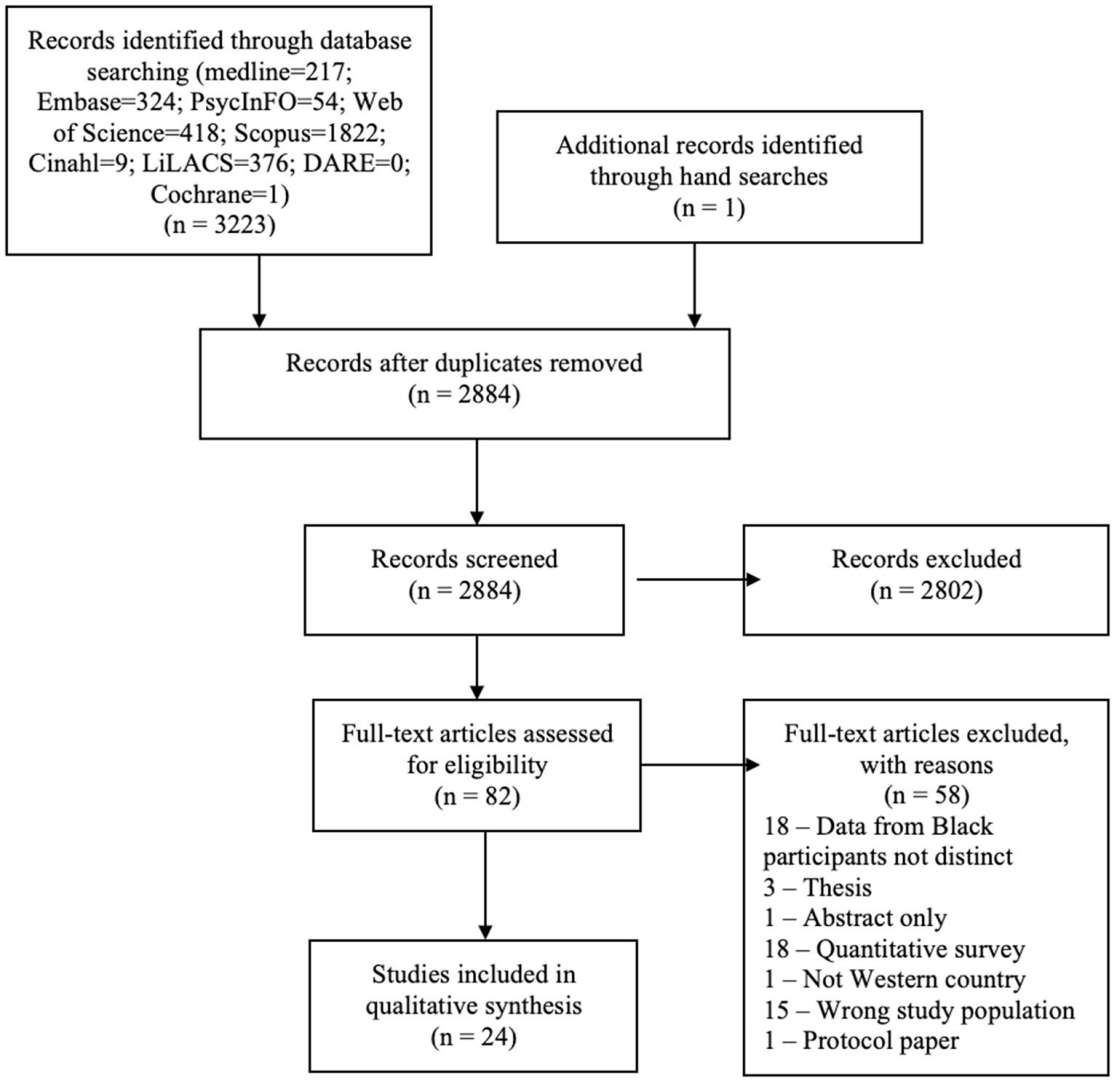
PRISMA diagram.

The search identified 3,223 titles (Figure 2). Following screening, 24 published papers were identified for inclusion in the review (Table 2). The search alert confirmed no further eligible papers were published between completion of the search and submission of the review for publication.

**Table 2:** Included studies.

**Study**	**Objective**	**Topic**
(38)	To understand how cultural factors influence perinatal outcome in Somalian women	General lifestyle
(39)	To examine barriers to physical activity in pregnancy	Activity
(40)	To explore perspectives on weight gain in women at risk of having a low birthweight baby	Weight and diet
(41)	To understand perceptions surrounding advice about diet and exercise in pregnancy	Diet and activity
(42)	To understand barriers and facilitators toward healthy lifestyle and weight	General lifestyle
(43)^a^	To understand views about physical activity and eating patterns in pregnancy	Diet and activity
(44)	To determine barriers to adoption of healthy diet in low-income pregnant women	Diet
(45)^a^	To gain insight into how low-income pregnant African American women view pregnancy weight gain	Weight
(46)	To understand perceptions of weight gain in urban, pregnant African American women	Weight
(47)	To explore cultural influences toward pregnancy and childbirth in Somalian women in Sweden	General lifestyle
(48)	To explore health beliefs of women who with a GDM diagnosis	General lifestyle
(49)	To understand beliefs about lifestyle during pregnancy in obese pregnant women	Weight
(50)^a^	To identify barriers and facilitators to exercise in pregnancy	Physical activity
(51)^a^	To explore attitudes of low-income African American women toward exercise in pregnancy	Physical activity
(52)	To identify reasons for late-night snacking	Weight
(53)	To explore attitudes and perceptions about GWG.	Weight
(54)	To describe the health experiences of low-income women who had GDM	General lifestyle
(61)	To explore migrant women's perceptions of health during pregnancy and PPartum	General lifestyle
(55)	To describe motivators and barriers to healthy eating in pregnancy	Diet
(56)	To explore attitudes toward weight gain and weight management interventions	Weight
(57)	To document the impact of a GDM diagnosis on East African immigrant women	General lifestyle
(58)	To understand factors influencing health in low-income postpartum women	General lifestyle
(59)	To understand attitudes toward risk of diabetes after GDM	General lifestyle
(60)	To describe perceptions of weight gain, activity, and nutrition during pregnancy	General lifestyle

a *Linked studies*.

### Description of Studies

Of the 24 studies included, 19 were conducted in America (39–46, 49–56, 58–60), 3 in Sweden (38, 47, 48), and 2 in Canada (57, 61). Eighteen studies concentrated on pregnancy (38–47, 49–53, 55, 57, 60), 4 on the postpartum period (54, 56, 58, 59) and 2 on both (48, 61). Five studies exclusively comprised a sample of women who had been diagnosed with GDM (47, 48, 54, 57, 59). Across the studies, 6 papers explicitly focused on weight (45, 46, 49, 52, 53, 55), 3 on physical activity (39, 50, 51), 2 on healthy eating (44, 55), and the remainder focused on a combination of these, or lifestyle in general. Qualitative data collection included focus groups (39, 41, 43, 45, 46, 49–52, 54, 56, 58), interviews (38, 40, 42, 44, 47, 48, 53, 55, 57, 59, 60). One study included ethnographic observation in addition to focus groups (61) (Table 2).

### Participant Characteristics

In total, data from 401 women were included in the analysis. The complete sample included women across all BMI categories. Six studies included only women who were overweight or obese (42, 49, 52, 53, 55, 58). Seven studies selected low-income women (43–45, 49–51, 58) and in 2 studies at least 80% of the sample were single mothers, although not chosen by purposive sampling (52, 53). Educational attainment was inconsistently reported across the studies but suggests a range of educational attainment levels. Two studies reported more than 50% of participants receiving tertiary education (41, 59) whilst five studies reported at least a quarter of participants had not proceeded beyond high school education (46, 52, 53, 55, 58). Seven studies included mixed ethnicities (39, 41, 47, 49, 54, 58, 59) and the remaining studies included only women of black African heritage. Five of the 24 studies identified the study sample as a first-generation migrant (38, 47, 48, 57, 61) and the remaining studies did not report generational status (Table 3).

**Table 3:** Study characteristics.

**Study**	**Sample and setting**						**Method**	
	**Country**	**Pregnancy stage**	**Ethnicity**	**Sample**	**Black**	**Weight**	**Theory specified**	**Data collection: analysis method (if specified)**
(38)	Sweden	P	A(East)^m^	15	All			Interviews; text analysis
(39)	US	P	A, C, H	40	48%	Mixed BMI		Focus groups
(40)	US	P	A	19^$^	All			Interviews
(41)	US	P	A, C, H	58	33%	Mixed BMI		Focus groups
(42)	US	P	A	33	All	Obese and Overweight		Interviews
(43)^a^	US	P	A	26^*^	All			Focus groups
(44)	US	P	A	25^*^	All			Interviews; content analysis
(45)^a^	US	P	A	26^*^	All			Focus groups
(46)	US	P	A	31	All		Grounded theory	Focus groups; grounded theory
(47)	Sweden	P	A(East)^m^ C	23^‡^	43%			Interviews
(48)	Sweden	P/PP	A^m^	9^‡^	All		Health belief model	Interviews; content analysis
(49)	US	P	A, H	16^*^	87%	Obese and Overweight	Health belief model	Focus groups; thematic analysis
(50)^a^	US	P	A	34^*^	All	Mixed BMI		Focus groups
(51)^a^	US	P	A	34^*^	All	Mixed BMI		Focus groups
(52)	US	P	A	18^†^	All	Obese and Overweight	Multiple	Focus groups
(53)	US	P	A	21^†^	All	Obese and Overweight	Grounded theory	Interviews; framework analysis, grounded theory
(54)	US	PP	A, H, Ap	24^‡^	33%			Focus groups
(61)	Canada	P/PP	A(East)^m^	80	All			Focus groups, observation; content analysis
(55)	US	P	A	21	All			Interviews
(56)	US	PP	A	22	All	Obese and Overweight		Focus groups
(57)	Canada	P	A(East)^m^	10^‡^	All			Interviews; content analysis
(58)	US	PP	A, C, H	25^*^^¶^	36%	Obese and Overweight		Focus groups; content analysis
(59)	US	PP	A, C, H	23^‡^	35%		Health belief model	Interviews; template analysis
(60)	US	P	A, C, H	30	All	Mixed BMI	Theory Planned behavior	Interviews

a Linked studies **Population characteristics:**

‡ *with GDM diagnosis*,

* *Low-income*,

$ *Teenage mothers*,

¶ *High risk for depression*,

† *80%+ single mothers; Ethnicity—A, Black African; C, Caucasian; H, Hispanic; A(East), East African; Ap, Appalachian; m, Migrant **Pregnancy stage**—P, Pregnancy; PP, postpartum*.

### Quality Appraisal

One study was considered to be of poor quality (40), one good (49) and the others fair, according to the CASP qualitative studies tool. The main reason for lower quality was an incomplete description of the data analysis process to indicate if it was sufficiently rigorous and lack of clarity in the statement of findings (Supplementary File 2).

### Study Methodology Comparisons

A comparison was made across the study methodologies (focus groups, interviews, and ethnographic studies) to compare coding and contribution to thematic analysis. Each methodology contributed equally to each of the themes. However, focus groups were preferentially used for discussions of weight and weight gain. This approach generated rich discussion and conclusions about the traditional attitudes and beliefs around weight and size, which were less evident from the interview data.

### Sensitivity Analysis

Sensitivity analyses were conducted to assess the impact on the finding of study quality (40) and contextual relevance to the review question (38, 39, 47, 48, 54, 57–59). After removing one study of poor quality, the sensitivity analysis did not alter the review findings.

### Findings of Thematic Synthesis

**Figure 3:**
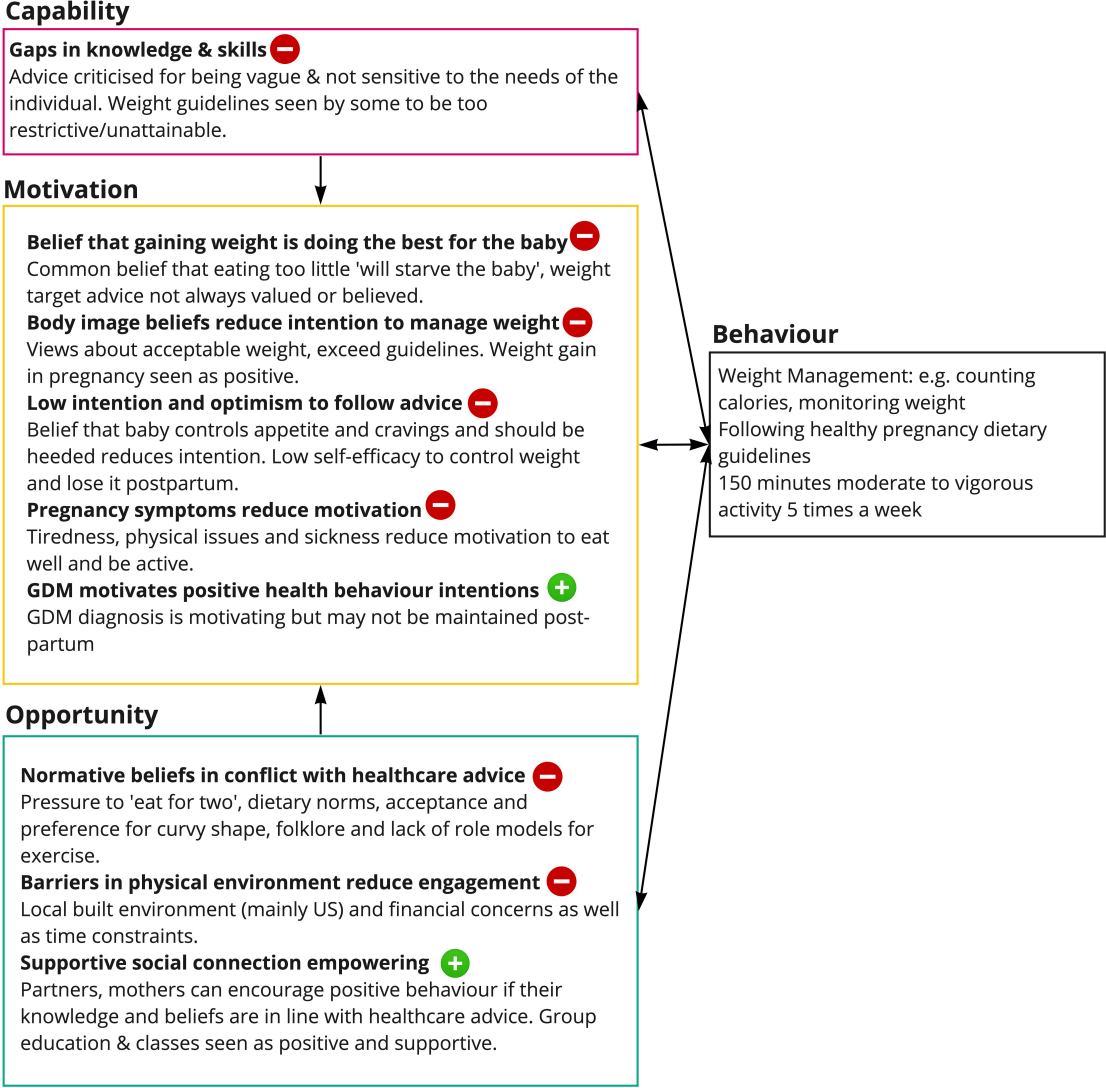
Summary of key themes from the COM-B analysis.

Across the domains of the COM-B framework, Motivation accounted for 54% of the coding, Opportunity for 37% (Social 28% and Physical 9%) and Capability for 9% (Figure 3).

The derivation of each theme from first and second-order constructs is detailed with verbatim examples in Supplementary File 4, together with details of the source publication. ***First-order constructs*** (participant quotes) are shown in bold italics and *second-order constructs* (researcher quotes) are shown in italics.

#### Capability

##### Psychological Capability

*Gaps in Knowledge and Skills.* Women felt healthcare advice was not as useful as it could be. Dietary advice was considered vague, negative and inconsistent, and it failed to take into account personal circumstances and cultural preferences (41, 49, 53, 57, 61). In terms of activity, women wanted to know specifically what type of exercise is safe in pregnancy and criticized health practitioners for not actively promoting physical activity (41, 50, 51). Krans et al. (50, 51) suggested that more practical advice on the duration, frequency, and the most appropriate type of exercise was needed. Gestational weight advice was generally seen as too restrictive, unattainable (46, 53, 56, 60) and inconsistent (49). However, a minority of women did find the gestational weight gain guidance useful (45, 60). Women said that to support postpartum weight loss they would like more information on calories, suggested meal plans and to learn how to cook traditional cultural foods in a healthy way (56).

#### Opportunity

##### Social Opportunity

*Beliefs of Community Networks Conflicted With Healthcare Advice.* Significant others offered advice that conflicted with that of healthcare practitioners (39, 40, 43, 45, 46, 49–53, 55). Pregnant women stated that they felt pressure from partners and family to “*eat for two*” creating conflict and resulting in stress for the mother-to-be and sometimes led to the advice being ignored (40, 44, 46, 49, 53). A social acceptance of larger body sizes and pressure from partners to retain their curvy shape resulted in women rejecting both BMI charts as an indicator of obesity and healthcare assertions of the need for weight management (45, 46, 49, 50).

Dietary norms within the community included regular consumption of fried foods, fast food and regular consumption of snacks. Women enjoyed the taste of these foods before pregnancy and consumption was perpetuated by others around them, making it difficult to change habits, even for those motivated to do so (40, 42, 43, 49, 50, 52, 56, 60). Multigenerational households were common; food choice was dictated by the primary homemaker and often did not align with recommendations (49).

Community beliefs, such as not raising your hands above your head while pregnant, created a fear of exercising during pregnancy (49, 51). In addition, there were reports of a lack of black cultural role models for women undertaking exercise in their everyday lives (50, 53).

*Supportive Social Connection Was Empowering.* The majority of studies reported that where family members, especially the pregnant woman's mother, understood healthy lifestyle guidance in pregnancy, it helped to improve compliance (39, 40, 42–44, 47–53, 55–58, 60, 61).

East African immigrant women described a “***we culture***” in their native countries. Community support meant being healthy while having a baby was a lot easier than in the HIC country (47, 61).

Building a relationship with the healthcare practitioner, feeling personally understood, and having personal circumstances acknowledged was important for women. This included practitioners tailoring advice to women's needs, understanding financial constraints, cultural dietary practices, difficulties attending appointments and being sympathetic to fluctuating motivation associated with pregnancy-related mood (41, 48, 53, 54, 56, 57, 61).

Mothers felt that learning, cooking and exercising in group classes would provide social support to help with weight loss (49, 50, 53, 56, 60). Postpartum women would like extended family to be able to join them, especially for nutrition and cooking sessions (56).

##### Physical Opportunity

*Barriers in the Physical Environment Reduced Engagement.* The physical environment, including local neighborhoods, workplaces, financial constraints, and lack of free time, were practical barriers to making positive lifestyle changes (39, 42–44, 47–50, 52, 54–58, 60, 61). In Sweden, the issues were primarily financial (47, 48). By contrast in the US and Canada, participants highlighted additional constraints of the built environment and food insecurity (39, 42, 50, 61). In the US studies, access to healthy food was described as limited while cheap unhealthy foods and fast-food shops were considered plentiful, perpetuating the consumption of less healthy alternatives (42, 44, 53, 55, 60, 61).

Healthy foods were seen as more expensive, limiting both the ability to eat healthily and limiting the desire to try new foods (44, 47, 52, 53, 55, 57, 60, 61). Women diagnosed with GDM expressed a strong need to comply with the guidance, but particular stress associated with the financial implications of doing so (47, 48, 57).

For East African women life “back home” in Africa was seen as more active, involving farming and walking, compared to life in Canada. In Canada, affordable facilities were not available and the weather was not conducive to being outdoors (61).

The need to juggle care of other children created barriers to exercise and made fast food an attractive option (39, 42–44, 49, 50, 56, 58, 60, 61).

#### Motivation

##### Reflective Motivation

*Gaining Weight Was Believed to be “Doing the Best for the Baby.”* Authors concluded women felt weight gain, “*no matter how much*” (45) was best for the baby, too little gain would “***starve the baby***” (46), that they were “***eating for two***” (53). Herring et al. (46) assert that “*few women listened to their obstetric providers about IOM recommended weight gain targets*”. This was echoed by comments from participants in other studies (45, 49, 60). Notably, little evidence is reported across the studies to indicate that women recognize the risks of GWG to the infant. Instead, they focus on the implications of weight gain on themselves (45, 60). Kroeger et al. explored what level of risk to the infant would curb unhealthy snacking. Women discussed issues such as “***birth defects or heart*** ***problems***” as being serious enough to motivate behavior change while having an obese child was not (52).

There were two notable exceptions to the main body of data. Young women at risk of having a low birthweight baby reported negativity toward weight gain and taking actions to ensure their body weight did not go up too much (40). A study of Somalian women (38) also described women restricting food intake to limit fetal growth which they felt would reduce the risk of a cesarean.

*Body Image Beliefs Reduced the Intention to Manage Weight.* Beliefs about body weight and what is attractive reduced the intention to manage weight. For example, Kominiarek *et al*. noted women “*described a body image that was not in line with standard clinical recommendations*” such as “***200 lbs. is not that big***” (49). One study reported pregnancy weight gain made women feel more attractive due to cultural preferences for a “***voluptuous***” shape (45). Extra weight “***in the right places***” (45) was welcomed, as long as it did not negatively impact on appearance or physical abilities (45, 51, 56).

*Women Had Low Intention an Optimism in Their Ability to Follow Advice.* Cravings are a key driver of dietary intake in pregnancy (40, 42–44, 46, 52–55, 60). Four of these studies suggest women believed that “*the baby is in control*,” and cravings and baby movements are linked to the baby communicating what it needs and that a pregnant woman should therefore respond to cravings (43, 46, 52, 55). Women described not having the desire to resist these cravings, which are often for “***tasty***” foods high in salt, fat and sugar (42, 43, 46, 52).

In general, women put off actively managing their weight during pregnancy but planned to do so after the birth whatever their starting weight (39, 45, 46, 51). However, women who enter pregnancy overweight or who have already had a child, show lower self-belief in their ability to do so (56, 59, 60).

*GDM Diagnosis Motivated Positive Health Behavior Intentions.* The GDM diagnosis gives women greater determination to control lifestyle in pregnancy (47, 48, 54, 57). However, Hjelm et al. (48) suggest that after pregnancy this motivation wanes.

##### Automatic Motivation

*Pregnancy Symptoms Reduce Motivation to Engage.* Tiredness, low energy, poor sleep, aches, pains, and mood swings reduced the desire to engage in physical activity in particular but also influenced increased appetite and snacking behavior (38, 39, 42, 43, 47, 49, 50, 52, 53, 55, 60).

### Behavior Change Technique Mapping

Following the COM-B thematic analysis, behavior change wheel (BCW) mapping was carried out to identify potentially useful evidence-based behavior change techniques, based on the synthesized data. The BCW suggests potential intervention functions for each of the COM-B domains. These include, for example, interventions designed to educate, to persuade or to restructure the environment. For each of these intervention functions, a range of evidence-based behavior change techniques are recommended. There is a large glossary of published behavior techniques (62), these are detailed and specific, e.g., providing information about health consequences: “*Provide information (e.g., written, verbal, visual) about health consequences of performing the behavior*” (32). These behavior change techniques can then be incorporated into the design of intervention components. For example, using educational videos as a component to provide information about health consequences.

**Figure 4:**
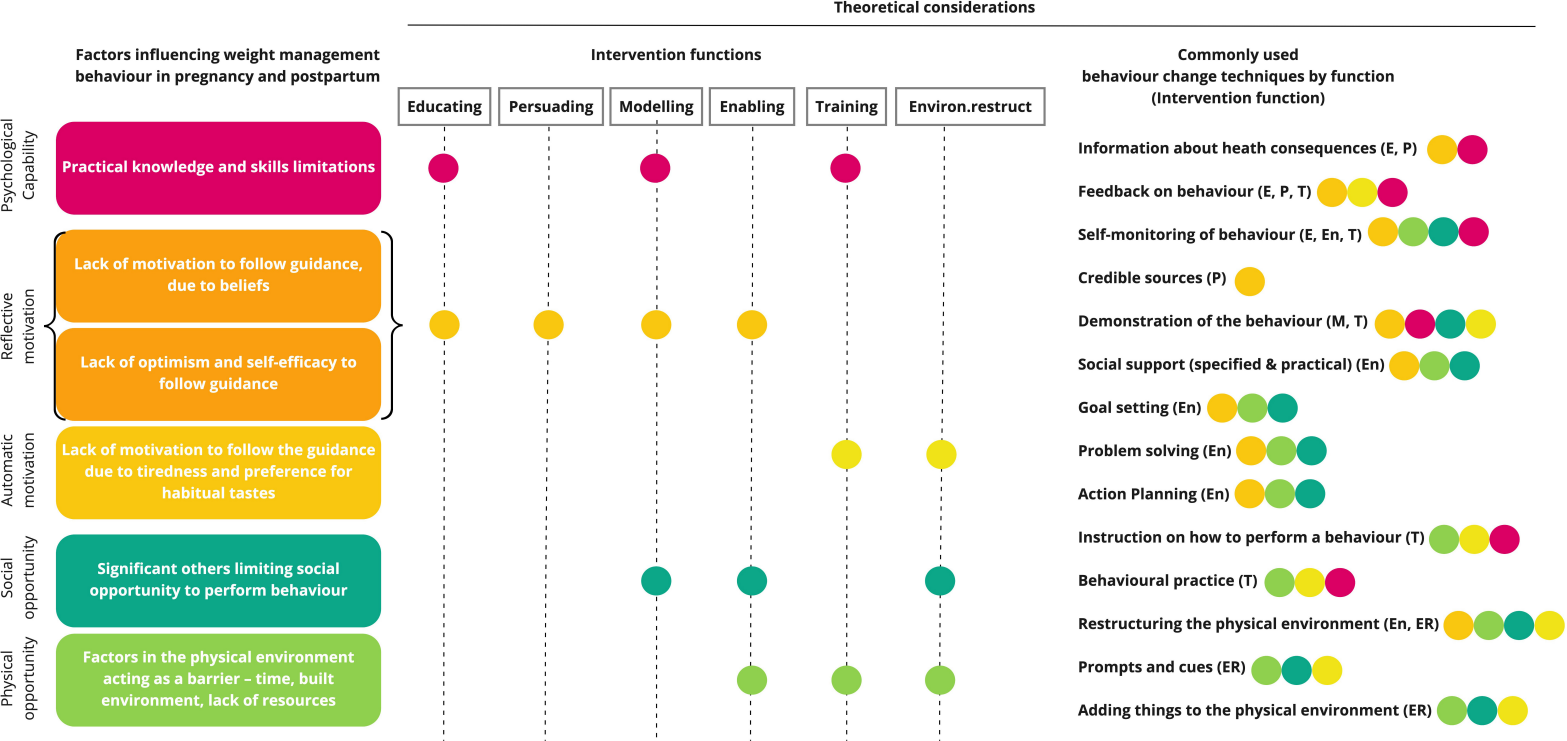
Behavior Change Wheel mapping.

Based on this approach, the mapping results are presented in Figure 4.

For motivational factors—for example reluctance to manage weight due to beliefs or lack of self-efficacy, interventions designed to educate, persuade, model, train, or enable are recommended. Potential behavior change techniques include *providing information about the health consequences* of weight gain in pregnancy, providing evidence from *credible sources*, which may translate into intervention components such as including peers or faith-leaders to promote the desired behavior. Techniques from control theory have also been shown to increase self-efficacy to perform health behaviors such as *goal setting, problem-solving*, and *action planning* (63). Barriers associated with automatic drivers, such as food cravings, may be supported by the techniques *adding things to the physical environment* or *providing instruction*. Intervention components such as providing healthy snacks or providing instruction about what snacks may be the best alternatives to the foods craved would support these techniques. Motivational factors may also be influenced by changes in Capability and Opportunity (Figure 1).

For social opportunity factors—such as barriers associated with normative beliefs and habits of significant others, interventions focused on modeling and enabling, are recommended. Given the suggestion in the data that social support was beneficial, *provision of social support (practical and specified)* may be beneficial. This could translate for example into including the baby's father in an educational intervention, to provide support to perform healthy behaviors. Modeling may include *demonstrating the behavior* in a practical way; for example, cooking classes would map well to the thematic findings.

Gaps in knowledge and skills may be supported by interventions designed to educate, model, and train. Behavior change techniques mapped to these intervention functions include *providing information about health consequences*. As the data showed, the majority of the women had little knowledge about the implication of weight on the health of the offspring. *Self-monitoring* may help keep weight more uppermost in the mind of the women and *demonstration of behavior*, such as working out calories in a portion size, can be incorporated as an intervention component into education classes.

Physical opportunity barriers are obviously more pervasive and in many cases demand *restructuring the environment*, to provide financial support or safe space to exercise. However, for factors such a time restriction associated with looking after other children, *practical social support* may be useful, such as the provision of accessible creche facilities.

## Discussion

The pregnancy and postpartum periods are potential intervention points to reduce long-term obesity risk as well as positively influence the health of offspring of women of African and Caribbean descent living in HIC. The review presents a synthesis of qualitative evidence, most of which comes from the US. Use of the theoretical COM-B framework suggests that in particular there are barriers in Motivation and Social Opportunity to performing healthy weight management behaviors, due to cultural beliefs and normative social influences. Analysis using the Behavior Change Wheel suggests that behavior change techniques such as the *provision of social support*, providing *information about health consequences*, use of *credible sources, demonstration*, and techniques from control theory e.g., *goal setting* are potential approaches to incorporate into intervention design.

Overall, the thematic synthesis indicated motivational barriers included beliefs contradictory to healthcare advice, such as maternal weight gain and responding to cravings being best for the baby and positive body image being associated with higher weight. Intention to manage weight *during* pregnancy was low, with women preferring to concentrate on weight management after the baby's birth; optimism in the ability to lose weight postpartum was low, particularly for women entering pregnancy overweight or obese. In addition, while social support was helpful for women, normative beliefs, and habits of significant others, driven by cultural norms could conflict with healthcare advice. This limited adherence to recommendations, even for those with positive intention. Additionally, there were evident gaps in knowledge about the risks of excessive weight gain to the fetus, the specific recommendations for physical activity and the translation of dietary advice to incorporate cultural preferences. Factors associated with the built environment such as the barriers associated with the density of fast-food and limited safe space to exercise were reported in the North American studies particularly. The five studies that specifically focus on migrant women highlight the challenges for women adjusting to the lack of support compared to “back home” (61) and the difficulties they faced due to a perceived lack of cultural saliency and healthcare sensitivity to their personal circumstances, especially the lack of financial resources (47, 57, 61). Gaps in knowledge were particularly evident amongst participants in these studies (38, 47, 48, 57) and were sometimes perpetuated by traditional cultural beliefs, such as limiting food intake to avoid a cesarean delivery (38) and stigma (57). Overall, contributions to the themes were broadly similar for migrant women and those of settled status, however three of the five studies focused on gestational diabetes and so exploration of attitudes to weight-related health behaviors was more superficial resulting in these studies contributing less to the summative findings.

The traditional cultural roots of the reported beliefs have been reflected in published literature. Studies amongst traditional African cultures identify an appreciation of a larger body size (64) and cultural taboos about the safety of exercise in pregnancy (65). The importance of responding to cravings has also been reported in West African communities; cravings were considered a signal from the fetus of what it needs and failure to respond to these signals was thought to harm the fetus (66). The presence of beliefs associated with positive benefits of pregnancy weight gain amongst women of African heritage and a low perceived risk of excessive gestational weight gain to the offspring, are supported by quantitative evidence (67). Conversely, barriers associated with tiredness and pregnancy symptoms are consistent with pregnant women in general, whatever their ethnicity (68).

Comparing the results of the analysis to current literature there is some evidence supporting the review findings. The provision of *social support* in lifestyle interventions, in terms of education delivered in a group setting or inclusion of a partner or family member, as part of lifestyle interventions, have been shown to be effective in African American studies (69–72). Faith-based interventions have been successful in the US setting for a number of health conditions, combining social support with endorsement from credible sources from pastors (73–76). Weight-management interventions in particular have proved successful in the faith setting (77, 78). COM-B analysis to support intervention design for type 2 diabetes education in UK African-Caribbean communities also suggested the potential of social support as a technique (79). While the gaps in knowledge were evident in the data, the access and provision of advice alone have not been conclusively shown to result in appropriate gestational weight gain (24, 80). However, the analysis suggested *demonstration* as a potential technique to explore, this approach could support the verbal and written advice provision; *demonstration* involves actively showing the behavior—such as exercise or cooking classes. In fact, Airhihenbuwa, who has contributed considerably toward understanding culturally tailored health promotion to support African Americans (81), explains how important this type of approach is, along-side written or pictorial health communication, because it sits well with the African oral traditions where seeing and listening are intertwined in knowledge development (82). The provision of *information about health consequences* may be helpful to improve understanding of the risks of excessive weight. However, behavior change theorists do advocate that this approach needs to be used concurrently with other techniques to increase self-efficacy, so the increased knowledge is accompanied with practical skills to alter outcomes (83). This would include use of behavior change techniques such as *goal setting, action planning*, and *problem-solving*, which have also been reported as useful in lifestyle interventions generally (63, 84). There is limited data evaluating restructuring of the physical environments as a behavior change technique, however epidemiological data suggest a link between poor accessibility to healthy foods and limited access to appropriate spaces for physical activity and excessive gestational weight gain and poorer pregnancy outcomes (80). Additionally, Physical opportunity to access to healthy foods, availability of safe spaces to exercise, and associated time and budgetary constraints all influence compliance with healthy lifestyle advice (85, 86).

COM-B and the Behavior Change Wheel framework have been developed from a synthesis of 19 theoretical frameworks across the discipline of behavior science (31). The approach has the advantage that it is an easily accessible, well-defined methodological approach, which can facilitate incorporating behavior change thinking into intervention design. However, this accessibility does, to a degree, belie the complexities of behavior science (28). In comparison, the Intervention Mapping framework, which also provides a step-by-step approach to intervention development, offers a more nuanced approach. While potentially this may lead to a more pertinent selection of behavior change techniques (87), it does require the integrated involvement of a behavior change scientist on the team, in order to inform the selection of theoretical constructs (88). As such, the approach of Michie et al. (89) may offer a pragmatic compromise to improve the mapping of theoretical constructs to behavior change techniques when a multi-disciplinary team is not an option.

This review synthesizes the available evidence in a novel and practical way to help inform intervention development to support weight-related health behaviors in pregnant and postpartum women of African heritage. The use of the COM-B framework and BCW allows researchers, health promotion planners and intervention designers to think more theoretically about how an intervention is intended to shift patterns in the performance of behavior and the data suggest potential behavior change techniques. However, while there is a reasonable body of data available and many of these factors are likely to be pertinent across the African diaspora, there is a discernable gap in the literature from across Europe. The transferability of findings to the European setting is reduced because of access issues to healthcare within the privatized medical sector in the US and because the dietary patterns of African American communities are more streamlined with the majority population than has been found in European settings such as the UK (90). In addition, it is also recommended that further participatory work would be done in any local community settings to improve salience of health promotion or intervention (26). It is also a limitation that with the exception of five studies, the majority do not report generational status, thus limiting an assessment of the influence of acculturating factors.

The findings of this review will be of particular value to healthcare providers, researchers, and designers of healthcare and health promotion interventions supporting this population. The review highlights the evidence to suggest that there are positive community assets, such as community social networks that may be leveraged to support healthful behavior and that practical demonstration and modeling of behavior may be particularly helpful to support positive change. The review also identifies the inequities and specific cultural barriers and beliefs which need to be considered. By identifying key determinants of behavior and theoretically associating these with evidence-based behavior change techniques, the findings can help contribute toward the theoretical understanding of pregnancy and postpartum weight-related health behavior in this population. The results may be of notable relevance for those supporting African American women.

## Conclusion and Future Directions

Synthesized qualitative evidence suggested that cultural beliefs and social norms shape weight-related health behavior during pregnancy and the postpartum period. This potentially reduces the intention and opportunity for women of African heritage living in HIC settings to engage in optimal health behaviors. Gaps in knowledge and practical environmental factors were also evident. Potential behavior change techniques such as *social support, demonstration, credible sources*, and techniques that can improve self-efficacy, e.g., *goal setting* are suggested by analysis using the Behavior Change Wheel. There is a discernable gap in the available literature from the European setting.

## Data Availability Statement

The original contributions presented in the study are included in the article/Supplementary Material, further inquiries can be directed to the corresponding author/s.

## Author Contributions

AM: study conception, search and selection, quality assessment, and drafting of the manuscript. AA: search and selection, drafting of the manuscript. AF: drafting of the manuscript. LG: study conception, search development, and drafting of the manuscript. CR: selection, quality assessment, and drafting of the manuscript. All authors contributed to the article and approved the submitted version.

## Conflict of Interest

The authors declare that the research was conducted in the absence of any commercial or financial relationships that could be construed as a potential conflict of interest.

## Footnotes

^1^See acknowledgments.

## Supplementary Material

Supplementary File 1 Details of the search strategy.

Supplementary File 2 Quality appraisal.

Supplementary File 3 Coding framework.

Supplementary File 4 Example verbatim quotes linking sources to themes.
