# Supplementary material for: Factors Influencing Pregnancy and Postpartum Weight Management in Women of African and Caribbean Ancestry Living in High Income Countries: Systematic Review and Evidence Synthesis Using a Behavioral Change Theoretical Model
Source: Front Public Health. 2021 Feb 17;9:637800. doi: 10.3389/fpubh.2021.637800 (PMC7925838; doi:10.3389/fpubh.2021.637800)
Supplement: Supplementary File 1 — Details of the search strategy. [file Table_3.DOCX]

Supplementary file 1: Search strategy Medline

| Sample/population |  |  |
| --- | --- | --- |
| Ethnicity | 1. | exp African Continental Ancestry Group/ or exp African Americans/ |
|  | 2. | exp Caribbean Region |
|  | 3. | West Indies/ |
|  | 4. | “black african”.mp |
|  | 5. | afro-caribbean.mp |
|  | 6. | Caribbean.mp |
|  | 7. | 1 or 2 or 3 or 4 or 5 or 6 |
| Life stage | 8. | exp pregnant women/ |
|  | 9. | exp Postpartum Period/ |
|  | 10. | pregnan*.mp |
|  | 11. | postnatal*.mp |
|  | 12. | antenatal*.mp |
|  | 13. | 8 or 9 or 10 or 11 or 12 |
| **Sample** | 14. | 13 and 7 |
| Phenomenon of Interest |  |  |
|  | 15. | exp food/ |
|  | 16. | exp diet/ |
|  | 17. | exp exercise/ |
|  | 18. | exp. physical fitness/ |
|  | 19. | exp Life Style/ |
|  | 20. | exp Sedentary Lifestyle |
|  | 21. | exp Obesity/ or obesity.mp |
|  | 22. | exp body image or body image.mp |
|  | 23. | body weight/ or exp body weight changes or exp weight gain or exp weight loss/ or exp overweight |
|  | 24. | Exp weight perception |
|  | 25. | exercise*.mp |
|  | 26. | physical activity*.mp |
|  | 27. | body image.mp |
|  | 28. | diet*.mp |
| Phenomenon | 29. | 15 or 16 or 17 or 18 or 19 or 10 or 21 or 22 or 23 or 24 or 25 or 26 or 27 or 28 |
| Design | 30. | exp “Surveys and Questionnaires”/ |
|  | 31. | exp interview/ |
|  | 32. | exp Focus Groups/ |
|  | 33. | “focus group”.mp |
|  | 34. | questionnaire*.mp |
|  | 35. | case stud*.mp |
|  | 36. | observ*.mp |
| Design | 37. | 30 or 31 or 32 or 33 or 34 or 35 or 36 |
| Evaluation | 38. | exp attitude to health/ |
|  | 39. | exp health knowledge, attitudes, practice/ |
|  | 40. | belief*.mp |
|  | 41. | view*.mp |
|  | 42. | experience*.mp |
|  | 43. | opinion*.mp |
|  | 44. | attitude*.mp |
|  | 45. | percept*.mp |
|  | 46. | feeling*.mp |
|  | 47. | understand*.mp |
| Evaluation | 48. | 38 or 39 or 40 or 41 or 42 or 43 or 44 or 45 or 46 or 47 |
| Research type | 49. | exp qualitative research/ |
|  | 50. | qualitative.mp |
|  | 51. | mixed methods.mp |
| Research type | 52. | 51 or 52 or 53 |
| Design/methods | 53. | 52 or 37 |
|  |  | 14 (population) and 29 (phenomenon) and 48 (evaluation) and 53 (design/research methods) |
