# Supplementary material for: Factors Influencing Pregnancy and Postpartum Weight Management in Women of African and Caribbean Ancestry Living in High Income Countries: Systematic Review and Evidence Synthesis Using a Behavioral Change Theoretical Model
Source: Front Public Health. 2021 Feb 17;9:637800. doi: 10.3389/fpubh.2021.637800 (PMC7925838; doi:10.3389/fpubh.2021.637800)
Supplement: Supplementary File 2 — Quality appraisal. [file Data_Sheet_1.PDF]

Supplementary File 2: Summary of CASP analysis

Study ID	Year	Clear Statement?	Qualitative Methodology?	Appropriate Design?	Recruitment Strategy?	Data Collection?	Relationships between participants?	Ethical Issues?	Data Analysis?	Clear Findings?	Value & relevance?	Overall Verdict
Essen	2000	✓	✓	✓	•	✓	•	✓	•	✓	•	Fair
Evenson	2009	✓	•	✓	•	✓	•	✓	✓	•	✓	Fair
Everette	2008	✓	•	✗	•	•	✗	•	✗	✗	✓	Poor
Ferrari	2013	✓	✓	•	•	✓	✓	✓	•	•	✓	Fair
Goodrich	2013	✓	•	•	•	•	•	•	•	•	✓	Fair
Groth	2013	✓	✓	✓	✓	✓	•	•	•	•	✓	Fair
Groth	2016	✓	✓	✓	✓	✓	•	•	•	•	✓	Fair
Groth	2012	✓	✓	✓	✓	✓	•	•	•	•	✓	Fair
Herring	2015	✓	•	•	•	•	•	•	•	•	•	Fair
Hjelm	2012	✓	✓	✓	•	✓	•	✓	✓	•	•	Fair
Hjelm	2018	✓	✓	✓	•	✓	•	✓	✓	✓	•	Fair
Kominiarek	2015	✓	✓	✓	✓	✓	•	✓	✓	✓	✓	Good
Krans	2011	✓	✓	✓	✓	•	•	✓	•	✓	✓	Fair
Krans	2012	✓	✓	✓	✓	•	•	✓	•	✓	✓	Fair
Kroeger	2019	✓	✓	•	✓	✓	✓	•	•	•	•	Fair
Nagourney	2019	✓	✓	•	•	✓	✗	•	•	✓	✓	Fair
Oza-Frank	2018	✓	✓	✓	✓	✓	•	✓	✓	✓	•	Fair
Quintanilha	2018	•	✓	✓	✓	•	✓	✓	•	✓	•	Fair
Reyes	2013	✓	✓	✓	•	✓	•	✓	•	✓	✓	Fair
Setse	2008	✓	✓	•	•	✓	•	•	•	✓	✓	Fair
Siad	2018	✓	✓	•	✓	✓	✓	✓	•	✓	•	Fair
Sterling	2011	✓	✓	•	•	✓	•	✓	•	•	•	Fair
Tang	2015	✓	✓	✓	✓	✓	•	✓	•	✓	•	Fair
Whitaker	2016	✓	•	•	✓	✓	•	✓	✓	✓	•	Fair

Good	✓
Moderate	•
Poor	✗

Full CASP questions available at https://casp-uk.net/casp-tools-checklists/

* Value and relevance is considered as relevance to the review question
