# Supplementary material for: Factors Influencing Pregnancy and Postpartum Weight Management in Women of African and Caribbean Ancestry Living in High Income Countries: Systematic Review and Evidence Synthesis Using a Behavioral Change Theoretical Model
Source: Front Public Health. 2021 Feb 17;9:637800. doi: 10.3389/fpubh.2021.637800 (PMC7925838; doi:10.3389/fpubh.2021.637800)
Supplement: Supplementary File 3 — Coding framework. [file Table_1.DOCX]

Supplementary File 3: Coding framework

| COM-B Domain | TDF | Coding |
| --- | --- | --- |
| Psychological CAPABILITY | Knowledge | Understanding of role of different foods, exercise & medication  Understanding of health guidance  Questions & gaps in understanding  Understanding of origins, causes and management of diabetes |
|  | Memory attention & decision processes | Planning and organisation |
|  | Behavioural regulation | Concentration, managing diet  Avoiding temptation and determination  Organising self & self-management planning |
| Physical CAPABILITY | Skills | Stamina to exercise  Ability to count calories |
| Social OPPORTUNITY | Social influences | Influence of church communities  Cultural beliefs and values  Cultural norms  Friends & family influences  Positive & negative influences from others |
| Environmental OPPORTUNITY | Environmental context & resources | Access to medical care  Time constraints  Facilities in local community (e.g commercial weight management programmes, gyms, outside space, shopping facilities)  Cost & financial  Roles & working lives |
| Motivation REFLECTIVE | Beliefs about capabilities | Attitudes and feeling about managing weight, diet and activity levels.  Fatalism and faith influences  Past successes & failures (weight management, regular exercise, dietary changes) |
|  | Intentions, goals | Belief in the value of weight loss/gain  Desire to manage long term health |
|  | Beliefs about consequences | Understanding of the risks of weight gain in pregnancy for mother and child  Understanding of the effects and benefits of lifestyle changes |
|  | Identity | Cultural identity  Role in family/community |
| Motivation AUTOMATIC | Reinforcement/ habit | Usual diet and lifestyle choices  Innate drivers e.g. hunger, cravings |
|  | Emotions | Feelings about weight & complications, diet and activity |
