# Supplementary material for: Factors Influencing Pregnancy and Postpartum Weight Management in Women of African and Caribbean Ancestry Living in High Income Countries: Systematic Review and Evidence Synthesis Using a Behavioral Change Theoretical Model
Source: Front Public Health. 2021 Feb 17;9:637800. doi: 10.3389/fpubh.2021.637800 (PMC7925838; doi:10.3389/fpubh.2021.637800)
Supplement: Supplementary File 4 — Example verbatim quotes linking sources to themes. [file Table_2.DOCX]

**Supplementary file 4: Translation of 1^st^ and 2^nd^ order constructs: Linking themes to sources**

| **3^rd^ order constructs** | **Label** | **Summary of 1^st^ & 2nd order constructs** | **Sources** | **Examples of verbatim ‘***first*’ **and ‘second*’* order quotes** |
| --- | --- | --- | --- | --- |
| **Capability (PC = psychological capability)** | | | | |
| **PC i: Gaps in Knowledge & Skills** | Gaps in knowledge & skills | There are evident gaps in knowledge around how to translate the guidelines into practice. This includes not understanding the specifics of the activity and dietary guidance and a mixed view of weight targets. When cultural foods are consumed, e.g. by East African women a lack of cultural relevance was noted. | (Everette 2008), (Krans 2011, 2012), (Oza-Frank 2018), (Setse 2008), (Whitaker 2016) | **‘They expressed the need for a better understanding of the calorie and fat content of different foods.** *‘How to count calories, because sometimes I get real confused about counting my carbohydrates...’ ‘I would like to deﬁnitely include diet as the most important thing......show me how to incorporate the food that I already eat, how to cook them healthy, how to eat the same things but just how to prepare them differently. I don’t wanna go on a strict diet regimen that I know I’m not gonna stick to long term.’***’.** (Setse 2008) |
|  |  | Mixed views are expressed about the value of healthcare advice and much of the advice is criticised for vagueness, creating gaps in knowledge and skills. Women seek further information outside the healthcare interaction. | (Essen 2000), (Everette 2008), (Ferrari 2013), (Groth 2012, 2013, 2016), (Herring 2012), (Hjelm 2011, 2018), (Kominiarek 2015), (Krans 2011, 2012), (Nagourney 2019), (Oza-Frank 2018), (Quintanilha 2012), (Said 2018), (Setse 2008), (Tang 2015), (Whitaker 2016) | ‘**A concern voiced particularly by overweight African American women was that providers’ recommendations were too general and not clearly applicable to them as individuals’**. (Ferrari 2013) |
| **Opportunity (SO = Social opportunity; PO = physical opportunity)** | | | | |
| **SO i: Normative beliefs in conflict with healthcare advice** | Clash between traditional beliefs and medical advice | Feeling conflicted between traditional beliefs about exercise/diet/weight and medical advice | Exercise – (Everette 2008), (Groth 2012),(Groth 2013), (Herring 2012), (Kominiarek 2015)  Diet – (Evenson 2009), (Everette 2008), (Goodrich 2013), (Groth 2013), (Herring 2012), (Kominiarek 2015), (Krans 2011), (Kroegar 2019), (Nagourney 2019), (Reyes 2013), (Setse 2008), (Whitaker 2016)  Weight – (Groth 2012), (Kominarek 2015), (Krans 2011, 2012), (Kroegar 2019), (Setse 2008) | ‘*One of the most common beliefs expressed was that if a pregnant woman raised her arms above the head, this would lead to the umbilical cord getting wrapped around the fetal neck, and risk strangling the fetus. This particular belief was perpetuated by family members and prevented many of the women from participating in a variety of activities including housework and from a variety of exercises including yoga and stretching*. ‘**Don’t be stretching your arms. You’re going to choke the baby. I was so scared to do it with my son. I kept going like this like oh damn. I can’t even stretch**’’. (Krans 2012) |
|  | Significant others hold the belief you should ‘eat for two’ | Feeling conflicted between traditional belief held by supportive others about ‘eating for two’ in pregnancy | (Everette 2008), (Groth 2013), (Nagourney 2019), (Kominiarek 2015), (Herring 2012) | **‘Like my grandma, everytime I go to her house, she be like, ‘you hungry? I got good foods. You have to feed that baby’. Even if you not hungry they try to feed you.** (Herring 2012) |
|  | Social dietary norms clash with health advice | Community dietary habits including a fast-food culture, regular meals eaten out and when cooking at home traditional cooking methods including a lot of salt and oil. | (Everette 2008), (Goodrich 2013), (Kroegar 2019), (Groth 2103), (Kominiarek 2015), (Krans 2011), (Setse 2008), (Whitaker 2016) | ‘**In the black community we are so used to cooking a certain way. Our grandparents cooked this way…’** (Setse 2008)  ‘**I love greasy food…but I think in my own mind that if I get away from the fried food and go to the healthy food, that I could… at least try to start to eat more, but by me having my auntie next door to me, and she – you know, she’s from down South. You know every Friday, they have a fish fry and the spaghetti and all that’.** (Kominiarek 2015) |
|  | Conflict between advice of mother and partner and health advice | Pregnant woman’s mother and her partners are key influencers of behaviour and their traditional beliefs either drive her behaviour or create stress if she is trying to follow guidance and it conflicts with her own mother’s advice. | (Herring 2012), (Kominiarek 2015) | **‘I’m your momma. I told you this—how this is supposed to be - and you’re gonna go over to this group and let them tell you something different? If your baby comes out like this, it’s your fault’’—you know, it’s just—having you confused, like—listen to my momma or go to the group?’.** (Kominiarek 2015) |
| **SO ii: Connecting to others – Being empowered by supportive social connection** | Culture back home more supportive | E.African immigrant women describe a ‘we culture’ back home, making it easier to follow lifestyle advice there, than in the Western country, both during pregnancy and in the postpartum period. | (Quantanilha 2012), (Hjelm 2011) | **‘Social support “back home” was commonly linked to women’s ability to eat healthy and be physically active, two health behaviours participants identified as key during the perinatal period. As examples, women highlighted that people in their communities would prepare foods they were craving or were special in their cultures and cook for them when they did not feel well or were recovering from childbirth. In relation to physical activity, women believed social support from their relatives and friends “back home” was a facilitator to being physically active’.** (Quantanilha 2012) |
|  | Support from family guides lifestyle behaviour | Where the pregnant woman’s mother and partner are supportive of activity it improves likelihood of the guidance being followed. The support of mother’s is particularly important, especially for the teenage mothers but across other age groups also.  Having other children can be encouragement to exercise (although adds to tiredness).  Not having encouraging support is seen as a barrier to adopting health lifestyle behaviours | (Evenson 2009), (Everette 2008), (Goodrich 2013), (Groth 2013, 2016), Hjelm 2011, 2018), (Kominiarek 2015), (Krans 2011, 2012), (Kroegar 2019), (Nagourney 2019), (Reyes 2013), (Setse 2008), (Siad 2018), (Sterling 2011), (Whitaker 2016) | **‘Many participants described emotional support and encouragement from family members and significant others to make healthy choices, such as family members cooking healthier foods, encouraging them to stay away from unhealthy foods and providing advice on healthy lifestyle choices. Partners played a particularly important role, as one participant described:** *‘My boyfriend is everything to me. Like everything I want to do, he’s like, “let’s do it”* **’.** (Nagourney 2019) |
|  | Group support is valued | Group education, cooking and exercise classes are seen as motivating, particularly if the women are at the same pregnancy or postpartum stage and of the same cultural background.  Women would like their extended family to be included in these groups. | (Kominiarek 2015), (Krans 2011), (Nagourney 2019), (Setse 2008), (Whitaker 2016)  (Kominiarek 2015), (Krans 2011), (Setse 2008) | **‘Support through programs that involve multiple patients with similar experiences would be a strategy to learn more about cooking, healthy eating, and would provide a social aspect to exercise or be physically active. These types of programs can also contribute to educating the patients’ families on ways they can be more supportive. For example, both patients and providers described a stronger support system when a pregnancy became complicated by gestational diabetes.’** (Kominiarek 2015) |
|  | Health care professional relating to you as an individual is valued | Women value a supportive relationship with their healthcare provider to support their health in pregnancy and postpartum. The relationship is seen as impersonal in some cases (Ferrari 2013), (Setse 2008). This includes relating to them as an individual, understanding the stages they are in (for example being aware that there will be periods of depression in the postpartum phase (Setse 2008)). It also includes giving them culturally relevant information and financially appropriate guidance (Oza-Frank 2018).  Women who had GDM in particular, wanted a healthcare partner (Oza- Frank 2018) | (Ferrari 2013), (Hjlem 2018), (Nagourney 2019), (Oza-Frank 2018), (Quintanilha 2012), (Setse 2008), (Siad 2018) | *‘I think the main important thing is for a health professional is to look at everyone. I don’t like to be put in a box. I think it works so much better when you work with me, my body fat, my body weight, my height, and not try to say that everyone my height should be a certain size.’* (Setse 2008)  **‘Communication issues with providers affected the quality and quantity of care received, including women’s knowledge, management, and follow-up of GDM’.** (Oza-Frank 2018) |
| **PO i: Experiencing environmental barriers** |  | Financial constraints limited food purchase and access to exercise classes and facilities. | (Groth 2016), (Hjelm 2011, 2018), (Krans 2011), (Kroegar 2019), (Nagourney 2019), (Oza-Frank 2018), (Quintanilha 2012), (Reyes 2013), (Setse 2008), (Siad 2018), (Whitaker 2016) | **‘Several mothers perceived healthy eating to be more expensive. Cost and taste converged when mothers described hesitation to try new foods because they could not be sure if they or their families would like the taste, citing the potential waste too high a cost. “When I’m in the supermarket, it’s too risky to buy it (healthy food) and then get it home and nobody likes it. ...I wanna try these different things, but I’m just so afraid to spend my money on it and then no one wants it’**. (Reyes 2013) |
|  |  | Neighbourhoods and workplaces perpetuated unhealthy dietary and activity habits. There was a lack of facilities to exercise and purchase healthy foods locally and the cost of transport meant that food on a day to day basis came from local shops and fast food outlets. | (Evenson 2009), (Goodrich 2013), (Groth 2016), (Kominiarek 2015), (Krans 2011), (Nagourney 2019), (Quintanilha 2012), (Reyes 2013), (Whitaker 2016) | ***‘*All of the women who participated in the focus group sessions lived in urban neighborhoods with minimal public resources for recreation and physical activity. Many women expressed frustration with a lack of parks, walking tracks and swimming pools where they could exercise or take their kids to exercise as a family. Facilities that were available to participants were either in a state of disrepair or had long waiting lists and strict membership requirements’.** (Krans 2011) |
|  |  | All women but especially those with other children, or who were single mothers struggled with finding time for exercise, or just simply for themselves, particularly if they were working. Lack of time also influenced eating habits, making fast food a more attractive option than cooking at home. | (Evenson 2009), (Goodrich 2013), Groth 2013, 2016), (Kominiarek 2015), (Krans 2011), (Quintanilha 2012), (Setse 2008), (Sterling 2011), (Whitaker 2016) | **‘the majority of women were single mothers. Several women discussed working long hours because they were the only financial support for their family and had very little personal time. These single mothers felt overwhelmed as the primary caregivers to their children, and they emphasized that they often struggled to bear all of the responsibilities normally shared between two parents. As a result, exercise was not seen as a priority during the day after working, childcare and housework’**. (Krans 2011) |
|  |  | Multigenerational households mean that it wasn’t always possible to choose what foods were purchased and sometime other members of the household would eat the pregnant woman’s healthy foods, like fruit. | (Groth 2016), (Kroegar 2019), (Reyes 2013) | *‘I actually was mad because I bought [fruit juice], and that’s baby juice, and somebody decided to drink it. And I didn’t want to make a big scene about it because we all pitch in. It’s like, ‘why my [fruit juice]? Out of everything?’ I will be like that for the healthy foods.’* (Reyes 2013) |
| **Motivation (RM = reflective motivation; AM = Automatic motivation)** | | | | |
| **RM i: Belief that gaining weight is doing the best for the baby** | Wanting to do the best for the baby | Mothers across the studies want to do the best for their baby. While they understand that they should be eating healthy and exercising and want to do the best for the baby, this does not always translate to appropriate behaviour. | Wanting to eat right for the baby – (Everette 2008), (Whitaker 2016), (Groth 2016), (Herring 2012), (Kominiarek 2015), (Kroegar 2019), (Nagourney 2019)  Knowing activity is beneficial – (Whitaker 2016), (Groth 2012), (Ferrari 2013), (Krans 2011, 2012) | *‘All participants stated that they wanted to eat a healthy diet during pregnancy. However, when asked to describe the types of foods they plan on eating during their pregnancy, one-third of women described a diet that did not meet dietary guidelines’.*(Whitaker 2016) |
|  | Believing weight gain central to the baby’s health | Women worried about too little weight gain being harmful for the baby, reducing birthweight and leading to prematurity. Researchers noted that there was little linkage between weight gain and negative implications for the baby. A study specifically focused on a group of mothers who ate unhealthy snacks at night (Kroegar 2019), concluded childhood obesity in the offspring did not concern mothers. | Weight gain is part of a healthy pregnancy -(Groth 2012), (Herring 2012), (Kominiarek 2015), (Kroegar 2019), (Reyes 2013)  Studies noting a lack of awareness of risks of GWG for the infant – (Groth 2012), (Whitaker 2016) | *‘These messages were grounded in the belief that eating was best for baby and that baby would not be harmed if mothers ate or gained too*  *much. However, too little weight gain might ‘starve the baby’ and lead to a series of negative consequences, including prematurity and low birth weight’.*(Herring 2012) |
| **RM ii: Body image beliefs reduce intention to manage weight** | Accepting and enjoying weight gain | Women are accepting of weight gain and subsequent weight retention as part of having a healthy pregnancy, therefore they don’t worry about it. Those who express concern about gain, still intend to put off thinking about weight until after pregnancy.  Some women expressed pregnancy gain as improving their body image. | Accepting of gain and subsequent weight retention – (Kominiarek 2015), (Kroegar 2019), (Groth 2012)  Now isn’t the time to worry about it – (Evenson 2009), (Groth 2012), (Herring 2012), (Kominiarek 2015)  Pregnancy weight improved body image – (Groth 2012) | **‘I just wanted a little booby!... When I was pregnant with my daughter, I just gained. I was a size 3, and I went to a size 9. And everybody was like, “Oh you look better that way, you look better that way!’.(**Groth 2012) |
| **RM iii: ‘I’m not the one in control” – Having a low self-efficacy to manage weight and follow lifestyle advice** | The baby is in control | The baby is in control and dictating food preferences and cravings. Movements are interpreted as the baby being hungry. | (Groth 2013), (Herring 2012), (Reyes 2013), (Kroegar 2019) | **‘I be in the restaurant and there be nothing I want. The baby be like, ‘mom no, not this’. I go somewhere else’** (Herring 2012)  **‘I would crave for something, but it’s not only me craving for it. The baby craves for it, too. So the baby wants fried chicken, I’m like, ‘Oh well.’ I can’t turn the fried chicken down, because if I eat something healthy, I might throw it up because the baby don’t like it.’.** (Reyes 2013) |
|  | Feeling powerless to resist cravings | Food choice is primarily driven by cravings, which women seem powerless to resist | (Everette 2008), (Goodrich 2013), (Groth 2013), (Groth 2016), (Herring 2012), (Kroegar 2019), (Nagourney 2019), (Oza-Frank 2018), (Reyes 2103), (Whitaker 2016) | **I crave things, that’s what makes it hard for me to stay to the vegetables and the fruits and things. And sometimes I slip and go get the bag of chips and the stuff and eat them ‘cause I’m craving it’.** (Goodrich 2013) |
|  |  | Describing a fast food habit, which is then difficult to change in pregnancy as those are the foods you crave. | (Groth 2013, 2016), (Kominiarek 2015), (Herring 2012), (Kroegar 2019), (Reyes 2013) | *‘Women reported frequently eating out in a variety of food establishments. The places they chose ranged from fast food eateries to take out, to family-style restaurants. Even when main meals were prepared at home, there was a tendency to daily obtain atl east one meal or snack from an outside source’.*  **‘I like fried food. I like more fried food, even though it’s not healthy, but now when I eat something that is baked I feel in my mind it’s not done. I don’t want it like this.’**  (Groth 2013) |
| **RM iv: GDM diagnosis motivates positive health behaviour intentions** | GDM diagnosis motivates | Women in the GDM studies report behaviour that suggests they are more motivated to follow lifestyle guidance. However, there are still practical barriers. | GDM - (Hjelm 2011, 2018), (Oza-Frank 2018), (Siad 2018) | **‘There seemed to be a tendency, from mostly following advice received during pregnancy to less compliance with advice over time, after deliver’.** (Hjelm 2018) |
|  | Worried about losing postpartum weight | Discussing the postpartum period women expressed frustration and concern about their ability to lose weight. Multiparous women suggested that not only was it hard to lose baby weight after the birth, they gain subsequent weight as well. | (Setse 2008), (Tang 2015), (Whitaker 2016) | *‘Several multiparous women expressed frustration about their failure to lose weight after their last pregnancy. Other multiparous women who had initially lost weight after the previous delivery verbalised frustration about not being able to maintain weight loss’.* **“After each kid I got bigger and bigger and it’s not just after each pregnancy, it’s like not only was the weight still there, I gained extra weight”.** (Setse 2008) |
| **AM i: Pregnancy symptoms reduce motivation to engage** | Pregnancy symptoms reduce motivation | Symptoms associated with pregnancy such as tiredness, aches and pains and sickness reduce desire to engage in physical activity and healthy eating. | General - (Essen 2000), (Evenson 2009), (Goodrich 2013), (Groth 2013), (Hjelm 2011), (Krans 2011), (Kroegar 2019), (Nagourney 2019), (Reyes 2013), (Whitaker 2016)  Mood swings – (Kominiarek 2015) | **‘The most commonly cited intrapersonal barrier to exercise was fatigue, especially in the 1^st^ and 3^rd^ trimesters. Women discussed how quickly they became fatigues when physically active, losing motivation for exercise’** (Goodrich 2013) |
